# Effect of Lemon Inhalation Aromatherapy on Postoperative Pain, Nausea and Vomiting, Delirium, and Inflammatory Markers After Coronary Artery Bypass Grafting: A Triple-Blind Randomized Trial

**DOI:** 10.5812/ijpr-168770

**Published:** 2026-02-10

**Authors:** Siavash Sangi, Seyed Mohammadreza Amouzegar Zavareh, Hedayat Sahraei, Mojtaba Sepandi, Alireza Moradi

**Affiliations:** 1 Student Research Center, School of Medicine, Baqiyatallah University of Medical Sciences, Tehran, Iran; 2 Department of Anesthesiology and Operation Room, School of Nursing and Midwifery, Shahid Beheshti University of Medical Sciences, Tehran, Iran; 3 Atherosclerosis Research Center, Clinical Sciences Institute, Baqiyatallah University of Medical Sciences, Tehran, Iran; 4 Neuroscience Research Center, Baqiyatallah University of Medical Sciences, Tehran, Iran; 5 Department of Epidemiology and Biostatistics, Faculty of Health, Baqiyatallah University of Medical Sciences, Tehran, Iran; 6 Student Research Committee, Faculty of Nursing, Baqiyatallah University of Medical Sciences, Tehran, Iran

**Keywords:** Postoperative Pain, Aromatherapy, Coronary Artery Bypass Grafting, Postoperative Nausea and Vomiting (PONV), Postoperative Delirium

## Abstract

**Background:**

Complementary and integrative medicine has gained increasing attention as a safe adjunct for improving postoperative outcomes, particularly in cardiac surgery where pain, postoperative nausea and vomiting (PONV), and delirium remain common and debilitating complications.

**Objectives:**

This randomized clinical trial aimed to investigate the effect of lemon inhalation aromatherapy on postoperative pain, PONV, delirium, and inflammatory markers in patients undergoing coronary artery bypass grafting (CABG) surgery.

**Methods:**

In this triple-blind, parallel-group randomized controlled trial, 104 adult patients scheduled for elective CABG surgery were randomly allocated to either lemon aromatherapy or an odorless placebo. Aromatherapy began the evening before surgery, was administered every two hours until anesthesia induction, and resumed continuously during mechanical ventilation and for 72 hours after extubation. Pain and PONV were assessed every 6 hours across eight postoperative shifts using a 0 - 10 Numeric Rating Scale. Delirium was evaluated at 24, 48, and 72 hours using the intensive care delirium screening checklist (ICDSC). Inflammatory markers [C-reactive protein (CRP), neutrophil-to-lymphocyte ratio (NLR)] were measured at baseline and 72 hours. Data were entered after double-checking into SPSS version 26. Continuous variables were tested for normality using the Shapiro-Wilk test; normally distributed variables were compared using independent *t*-tests, and non-normal variables with the Mann-Whitney U test. Categorical variables were analyzed using chi-square or Fisher’s exact test. All analyses were two-tailed with a significance level of P < 0.05.

**Results:**

Of the 104 randomized patients, 103 completed the study (one patient in the intervention group was withdrawn due to postoperative cerebrovascular insult). The two groups were well-balanced at baseline with respect to demographic and clinical characteristics. Pain scores were significantly lower in the intervention group at all eight postoperative shifts (P < 0.001). Postoperative nausea and vomiting severity was significantly reduced across all shifts (P < 0.001), with fewer vomiting episodes and lower need for rescue antiemetic medications. The incidence of delirium was significantly lower in the intervention group at 24 hours (11.8% vs. 28.8%), 48 hours (15.7% vs. 34.6%), and 72 hours (7.8% vs. 23.1%) (all P < 0.05). Significant improvements were also observed in multiple ICDSC subscales, particularly inattention, disorientation, sleep-wake disturbances, and inappropriate speech/mood. At 72 hours, both CRP and NLR levels were significantly lower in the aromatherapy group, suggesting attenuation of the postoperative inflammatory response. No aromatherapy-related adverse events were observed.

**Conclusions:**

Lemon inhalation aromatherapy was effective in significantly reducing postoperative pain, PONV, delirium incidence, and inflammatory markers following CABG surgery. As a safe, low-cost, and non-invasive complementary intervention, lemon aromatherapy may enhance postoperative recovery and represents a promising adjunct to standard care in cardiac surgery.

## 1. Background

Coronary artery disease (CAD) is the leading global cause of morbidity and mortality, driven by lifestyle changes and aging populations (1, 2). Coronary artery bypass grafting (CABG) is a well-established intervention that significantly improves survival and quality of life in patients with severe CAD (2). Despite declining mortality rates following CABG, major complications persist in approximately 41% of patients (3). Globally, CAD accounts for 17.3 - 18.6 million deaths annually (4, 5).

Postoperative delirium is among the most prevalent and debilitating neurological complications following CABG, with incidence rates of 18 - 50% (6). This transient disorder of attention and cognition, unexplained by preexisting conditions, may exceed 90% in cases involving cardiopulmonary bypass (CPB) and hypothermia (7). This condition is associated with increased mortality (OR = 1.95), prolonged hospitalization (by an average of 2.2 days), long-term cognitive impairment, and reduced one-year survival (8). Similarly, postoperative nausea and vomiting (PONV) persist as major challenges; over 70% of CABG patients experience moderate-to-severe pain and approximately one-third suffer from PONV following general anesthesia, especially with opioid exposure (9-11). These complications prolong recovery, delay discharge, and increase healthcare costs (12).

Given these multifactorial postoperative challenges, non-pharmacological complementary interventions have received growing attention for their safety and effectiveness in perioperative care (13). Among complementary approaches, aromatherapy has shown promise in reducing pain, anxiety, fatigue, and sleep disturbances, thereby improving recovery and quality of life (14, 15). This olfactory-based therapy utilizes essential oils from plants such as lavender, orange, bergamot, rose, and lemon (15, 16). Studies on aromatherapy have demonstrated that the molecular constituents of essential oils and plant-derived phytochemicals can exert notable influences on human emotional responses (17). The literature indicates that aromatherapy is a pivotal strategy in diverse health fields including surgery, geriatrics, mental health, obstetrics, and pediatrics, and is commonly used in dialysis to ease neuropathic pain in renal patients (18).

Lemon (*Citrus limon*) essential oil, rich in limonene and citral, possesses antioxidant, anti-inflammatory (19), analgesic (20), antibacterial, and anxiolytic properties (15). It modulates serotonin pathways, thereby reducing nausea and vomiting (21), and has been shown to exert sedative and antidepressant effects in animal studies (20) as well as hypotensive and cytoprotective actions contributing to analgesia (19). Despite these promising findings, previous studies have reported inconsistent results regarding the physiological and postoperative effects of lemon aromatherapy (15). 

## 2. Objective

Given the high prevalence and adverse consequences of delirium, pain, and PONV after CABG, and the need for safe, affordable adjunctive therapies, this study evaluated the effect of lemon inhalation aromatherapy on these outcomes in patients undergoing CABG at Baqiyatallah University of Medical Sciences.

## 3. Methods

### 3.1. Design

This prospective, single-center, triple-blind, parallel-group randomized controlled trial was registered in the Iranian Registry of Clinical Trials (IRCT20231021059799N2; approval date: 17 June 2025; ID: 83709). The trial protocol was developed and reported in accordance with the CONSORT 2010 guidelines to ensure methodological transparency and reproducibility.

### 3.2. Settings

The study was conducted among adults scheduled for elective CABG at Baqiyatallah University of Medical Sciences. All assessments took place in the Cardiac Surgery Department, the Open-Heart Intensive Care Unit (ICU), and affiliated cardiac care units under standardized clinical protocols.

### 3.3. Sample Size

Based on our pilot data (n = 20; mean ± SD= 1.7 ± 2.6; Cohen’s d = 0.65) and validation from previous studies (15), we determined the sample size for the primary outcome (pain severity) using G-Power 3.1. For a two-sided *t*-test (α = 0.05, power = 80%), 68 participants were required. With a 20% attrition adjustment, this increased to 85 (43/group). To ensure ≥ 80% power for delirium incidence (co-primary outcome), we assumed a reduction from 32% (control) to 15% (intervention) based on pilot data and previous studies (15). This required 98 participants. Thus, 104 participants (52/group) were recruited, providing 86.4% power for pain severity and 82.1% power for delirium detection (α = 0.05, two-sided Fisher’s exact test).

### 3.4. Eligibility Criteria for Participants

**Figure 1.**
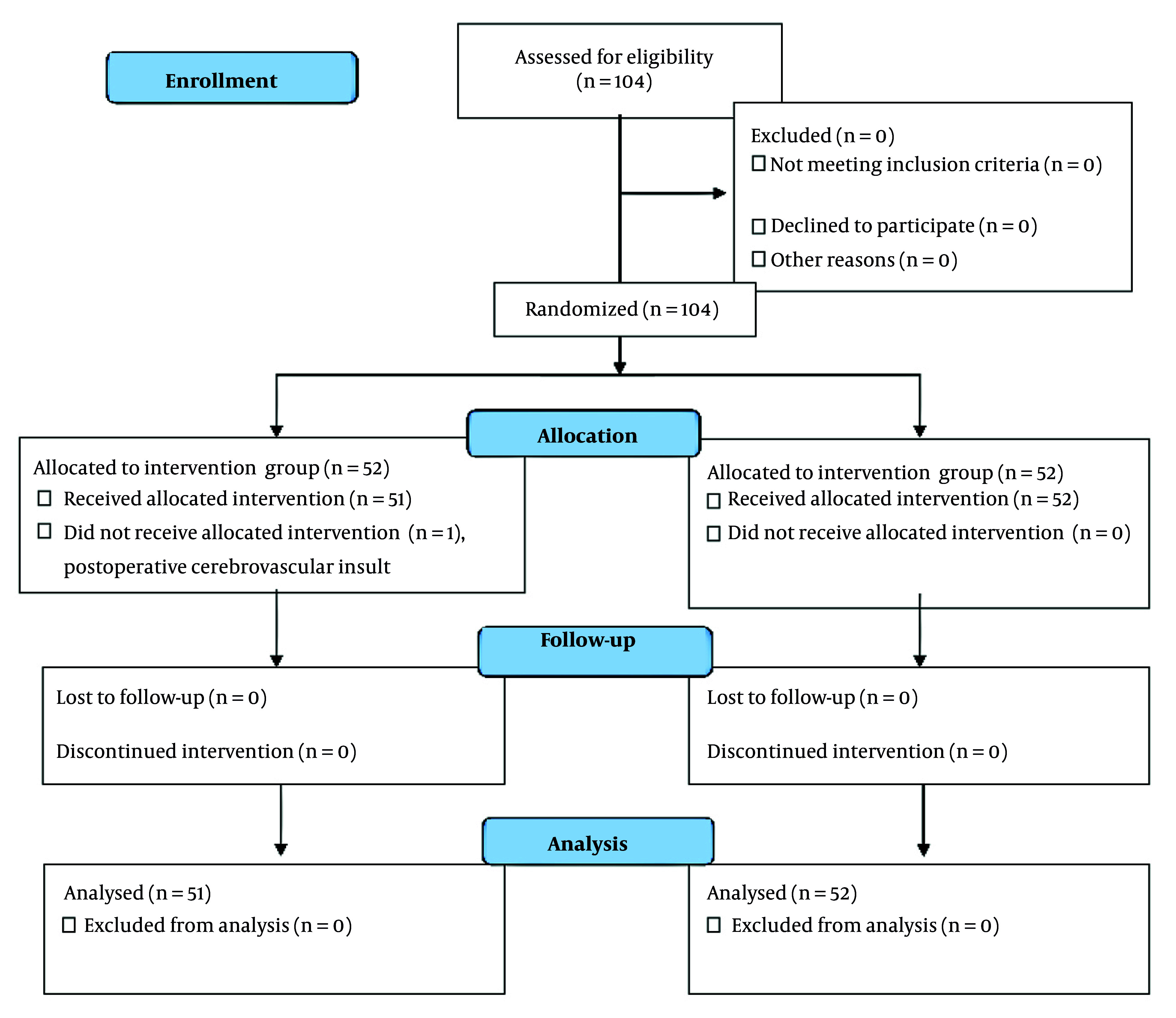
CONSORT flow diagram of participants in this study

Participants were consecutively recruited during the study period. Inclusion criteria were established to ensure sample homogeneity and to target individuals at elevated risk for the outcomes of interest. Eligibility required adults aged 40 - 80 years with an American Society of Anesthesiologists (ASA) physical status of class I to IV. Additional inclusion criteria included literacy, orientation to time/place/person, and intact verbal communication at the time of consent. Exclusion criteria included participation in similar trials, a personal or family history of postoperative delirium, and the presence of active psychiatric disorders such as major depression, anxiety disorders, or psychosis. Additional exclusion criteria included active postoperative bleeding requiring transfusion, excessive sedative or analgesic requirements beyond ICU protocol, prolonged surgical duration, the need for reoperation, known allergies to herbal preparations, and any condition that impaired olfactory function or nasal patency. Of 104 enrolled patients, one in the intervention group died postoperatively from cerebrovascular insult, leaving 103 for final analysis (51 intervention, 52 control; Figure 1). 

### 3.5. Randomization

An independent statistician generated the randomization sequence using permuted blocks of four in Microsoft Excel 365. Allocation concealment was maintained using sequentially numbered, opaque, sealed envelopes. After consent and confirming eligibility, envelopes were opened by a research nurse in the presence of a witness. Group allocation was disclosed solely to the anesthesia specialist responsible for preparing and labeling the coded aromatherapy solutions. Patients, clinical staff, outcome assessors, and the data analyst remained blinded until the database was locked. Post-randomization baseline characteristics were comparable (all P > 0.3), and the Berger-Exner test confirmed the absence of selection bias (reverse cumulative OR = 1.05, 95% CI: 0.79 - 1.39, P = 0.74), providing strong evidence that allocation concealment was successfully maintained and selection bias was effectively prevented.

### 3.6. Blinding Procedures

This triple-blind design masked participants, clinical care providers, and data analysts from group allocation. Participants were informed that they would receive inhalation of a neutral, pleasant aroma as part of their postoperative care, without disclosure of the specific essential oil used. Interventions were delivered via indistinguishable, code-labeled bottles prepared by an independent anesthetist, ensuring no discernible differences in appearance, odor intensity, or packaging between lemon and bitter almond oils. Bitter almond oil was selected as the control due to its minimal odor; both oils were identically diluted and administered (Barij Essence Pharmaceutical Co., Iran). Outcome evaluations, encompassing pain, nausea/vomiting via Numeric Rating Scale (NRS), and delirium via intensive care delirium screening checklist (ICDSC), were conducted by blinded ICU staff. Data entry into SPSS utilized anonymized codes, with group identities unmasked only after the analysis was complete. This approach, aligned with CONSORT recommendations, robustly preserved trial integrity and objectivity.

### 3.7. Inhalation Aromatherapy Intervention and Control Group Protocol

**Figure 2.**
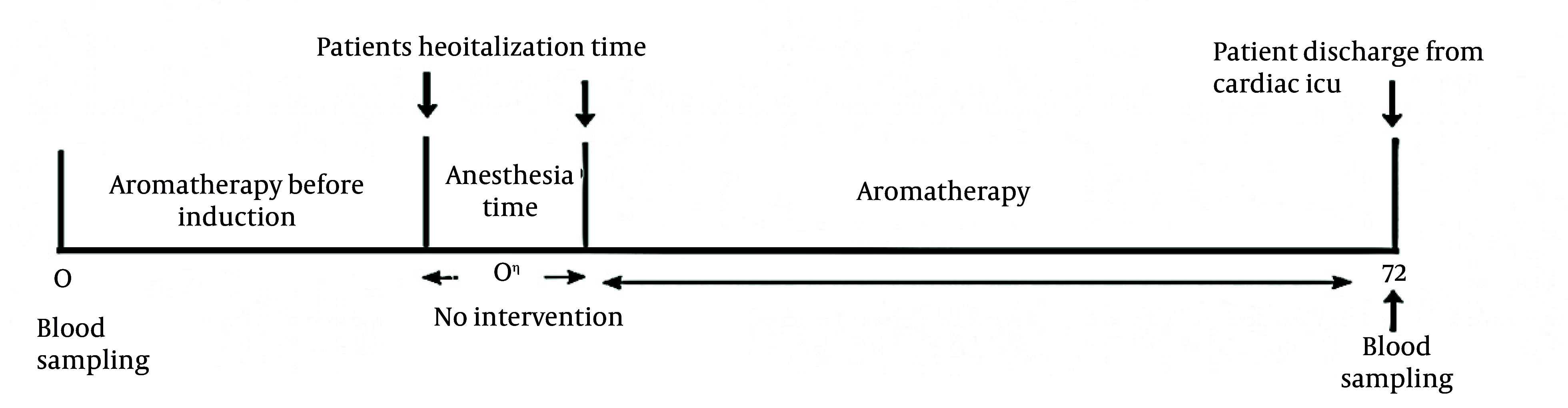
Time line of this study

The intervention consisted of controlled inhalation of lemon essential oil (*C. limon*) administered according to a standardized protocol. Lemon essential oil was selected based on prior evidence supporting its anxiolytic, analgesic, and antiemetic properties (13, 15). The procedure was conducted according to a standardized protocol to ensure consistency across participants. In the intervention group, five drops of lemon essential oil (Barij Essence Pharmaceutical Co., Iran) were applied to a sterile cotton pad attached to gauze and positioned approximately 10 cm from the patient’s nose. The intervention began on the evening prior to surgery and was reapplied every two hours until the induction of anesthesia. The intervention was paused during surgical anesthesia and CPB. As shown in our timeline (Figure 2), upon transfer to the ICU while intubated, aromatherapy resumed by placing the essential oil-saturated cotton pad within a 40 μm mesh enclosure in the external oxygen flowmeter tubing (4 L/min), positioned 15 cm upstream from the ventilator-humidifier interface with a dedicated bacterial filter separating it from the breathing circuit. Hourly circuit integrity checks confirmed no contamination or adverse respiratory events occurred. Postoperatively, aromatherapy was resumed immediately after the return of spontaneous breathing and continued for 72 hours following extubation (or until discharge if earlier). During mechanical ventilation, the cotton pad was placed within the oxygen mask tubing to ensure continuous exposure. The control group received an identical protocol using a low-odor bitter almond essence matched in volume and delivery schedule to mimic the sensory experience without active aromatic components. Both essences were placed in identical, coded bottles labeled as “Solution A” and “Solution B”, prepared and blinded by an independent researcher not involved in patient recruitment or data collection. Neither the participants, clinical staff, nor outcome assessors were aware of the group allocations throughout the study. The lemon essence used in this study was verified and certified by the quality control unit of Barij Essence Pharmaceutical Co. Its composition was analyzed using gas chromatography–mass spectrometry (GC-MS, Agilent 6890), confirming major constituents such as limonene, β-terpinene, γ-terpinene, β-caryophyllene, neral, α-terpineol, neryl acetate, geranial, and geranyl acetate. Previous studies have reported no adverse reactions or phototoxic effects associated with this product (22), and participants were informed of the minimal risk of irritation. All patients were instructed to report any discomfort, and the intervention would be discontinued immediately in the event of adverse effects. This rigorous and standardized approach ensured methodological precision and enhanced the reliability of the study outcomes.

### 3.8. Anesthesia Protocol for Coronary Artery Bypass Grafting Patients

Baseline vital signs were recorded by blinded anesthesia specialists. All patients received standardized anesthesia: Induction with propofol (1 - 2 mg/kg), midazolam (0.05 - 0.5 mg/kg), fentanyl (2 - 10 mcg/kg), and atracurium (0.5 mg/kg); maintenance via uniform infusion of fentanyl, atracurium, and midazolam. Ventilation was standardized to a tidal volume of 6 - 8 mL/kg to minimize confounders. Maintenance anesthesia was uniform across patients via infusion of three ampoules each of fentanyl, atracurium, and midazolam in a 50-mL syringe at 10 - 15 mL/min. Intraoperative monitoring included invasive blood pressure (IBP), pulse oximetry (POM), electrocardiogram (EKG), Bispectral Index (BIS), central venous pressure (CVP), and temperature. Activated clotting time was maintained above 480 seconds with heparin (300 - 400 IU/kg), reversed at procedure end with protamine sulfate (1 mg per 100 IU heparin). Invasive monitoring and ventilation continued in the cardiac ICU, with uniform environmental conditions (lighting, temperature, noise).

### 3.9. Outcome Measures and Data Collection Procedures

Outcomes were assessed using validated instruments to ensure reliability and reproducibility of the measured effects in CABG patients. Primary endpoints included postoperative delirium, pain intensity, and PONV incidence. The intervention commenced the night before surgery, administered by a blinded nurse and renewed every two hours until anesthesia induction. It paused during surgery; upon transfer to the ICU while still intubated, 5 drops of lemon essential oil were added to the flowmeter at 4 L/min for the intervention group. After extubation, it continued on a pad for 72 hours. Controls received odorless bitter almond oil via the same protocol. Delirium was assessed at 24, 48, and 72 hours postoperatively using the ICDSC. Each item is scored based on observations made over an 8-hour shift, with seven items rated as either absent (0) or present (1), and level of consciousness categorized into five levels (23). A total score of 4 or greater is indicative of delirium. The reliability of the Persian version of this checklist is approved by Cronbach’s alpha = 0.85 (24). All screenings were by group-blinded nurses. Delirium assessment followed the Society of Critical Care Medicine (SCCM) guidelines (25): Initial screening used the ICDSC (score ≥ 4 defined screen-positive), with immediate confirmation of positives via confusion assessment method for the ICU (CAM-ICU) only for ICDSC-positive cases by independent assessors blinded to group allocation. This two-step protocol optimizes diagnostic accuracy in intubated cardiac patients. Pain and PONV were measured every six hours using a standardized 0 - 10 NRS (0 = no sensation, 10 = worst imaginable), with PONV also noting vomiting frequency, volume, and duration. Higher scores indicate a higher intensity of nausea and vomiting. In this study, the reliability of the index using Cronbach’s alpha was approved (0.93). These measures captured the intervention's role in enhancing post-CABG comfort and recovery.

### 3.10. Laboratory Investigations and Biomarker Analysis

To probe anti-inflammatory mechanisms, venous blood (10 mL) was drawn at admission and ICU discharge, centrifuged for serum storage at -80°C, and analyzed blindly by certified technicians. C-reactive protein (CRP) and neutrophil-to-lymphocyte ratio (NLR) assessed inflammatory changes; elevated postoperative levels correlate with delirium and adverse cardiac outcomes. Figure 2 outlines the patient timeline.

### 3.11. Data Management and Statistical Analysis

Data were double-checked for accuracy and analyzed using SPSS version 26. Normality of continuous variables was examined using the Shapiro-Wilk test. Normally distributed variables were analyzed with independent t-tests, whereas non-normal variables were compared using the Mann-Whitney U test. Categorical variables were compared using chi-square or Fisher’s exact test. All analyses were two-tailed, and a significance level of P < 0.05 was considered statistically meaningful.

## 4. Results

Of the 104 randomized patients, 103 completed the trial. One participant in the intervention group was withdrawn due to a postoperative cerebrovascular event. Baseline demographic and clinical characteristics were comparable between the two groups, with no statistically significant differences observed (all P > 0.30). Detailed baseline characteristics are presented in Table 1. No statistically significant differences were observed in any variable.

**Table 1.** Baseline Characteristics of Control and Intervention Groups ^a^

Variables	Control (N = 52)	Intervention (N = 51)	Test Statistic (χ²)	P-Value
**Age (y)**			χ² = 2.55	0.466
40 - 50	5 ± 9.6	7 ± 13.7)		
50 - 60	17 ± 32.7	10 ± 19.6)		
60 - 70	20 ± 38.5	21 ± 41.2)		
≥ 70	10 ± 19.2	13 ± 25.5)		
**Gender**			χ² = 0.23	0.629
Male	33 ± 63.5	30 ± 58.8)		
Female	19 ± 36.5	21 ± 41.2)		
**Marital status**			χ² = 0.34	0.563
Single	7 ± 13.5	5 ± 9.8)		
Married	45 ± 86.5	45 ± 90.2)		
**Education level**			χ² = 2.67	0.445
Illiterate	9 ± 17.3	8 ± 15.7)		
< Diploma	13 ± 25.5	19 ± 37.3)		
Diploma	15 ± 28.8	15 ± 29.4)		
University	15 ± 28.8	9 ± 17.6)		
**Occupation**			χ² = 0.82	0.845
Unemployed	14 ± 26.9	16 ± 31.4)		
Employee	14 ± 26.9	11 ± 21.6)		
Worker	14 ± 26.9	12 ± 23.5)		
Self-employed	10 ± 19.2	12 ± 23.5)		
**Prior hospitalization**			χ² = 4.64	0.046
No	19 ± 36.5	9 ± 17.6)		
Yes	33 ± 63.5	42 ± 82.4)		
**Prior ICU admission**			χ² = 3.52	0.075
No	34 ± 65.4	24 ± 47.1)		
Yes	18 ± 34.6	27 ± 52.9)		
**Hypertension**			χ² = 0.08	0.780
No	34 ± 65.4	32 ± 62.7)		
Yes	18 ± 34.6	19 ± 37.3)		
**Diabetes**			Fisher's exact	0.608
No	41 ± 78.8	38 ± 74.5)		
Yes	11 ± 21.2	13 ± 25.5)		
**Renal failure**			χ² = 0.16	0.821
No	38 ± 73.1	39 ± 76.5)		
Yes	14 ± 26.9	12 ± 23.5)		
**Liver failure**			χ² = 0.50	0.715
No	47 ± 90.4	48 ± 94.1)		
Yes	5 ± 9.6	3 ± 5.9)		
**Smoking**			χ² = 0.28	0.594
No	42 ± 80.8	39 ± 76.5)		
Yes	10 ± 19.2	12 ± 23.5)		
**Addiction**			χ² = 0.36	0.618
No	51 ± 98.1	49 ± 96.1)		
Yes	1 ± 1.9	2 ± 3.9)		
**ASA class**			Fisher's exact = 1.44	0.818
I	0 ± 0.0	1 ± 2.0)		
II	3 ± 5.8	4 ± 7.8)		
III	31 ± 59.6	27 ± 52.9)		
IV	18 ± 34.6	19 ± 37.3)		
**BMI (kg/m²)**	25.45 ± 5.04	26.93 ± 3.94)	*t* = -1.66	0.101
**Surgery duration (min)**	105.51± 16.94	109.57 ± 24.34)	*t* = -0.99	0.327
**CPB duration (min)**	86.92 ± 13.67	88.98 ± 16.68)	*t* = -0.69	0.492
**Cross - clamp duration (min)**	67.69 ± 12.17	69.22 ± 15.54)	*t* = -0.56	0.578

Abbreviation: ICU, intensive care unit.

^a^ Values are expressed as mean ± SD.

### 4.1. Primary Outcomes

**Figure 3.**
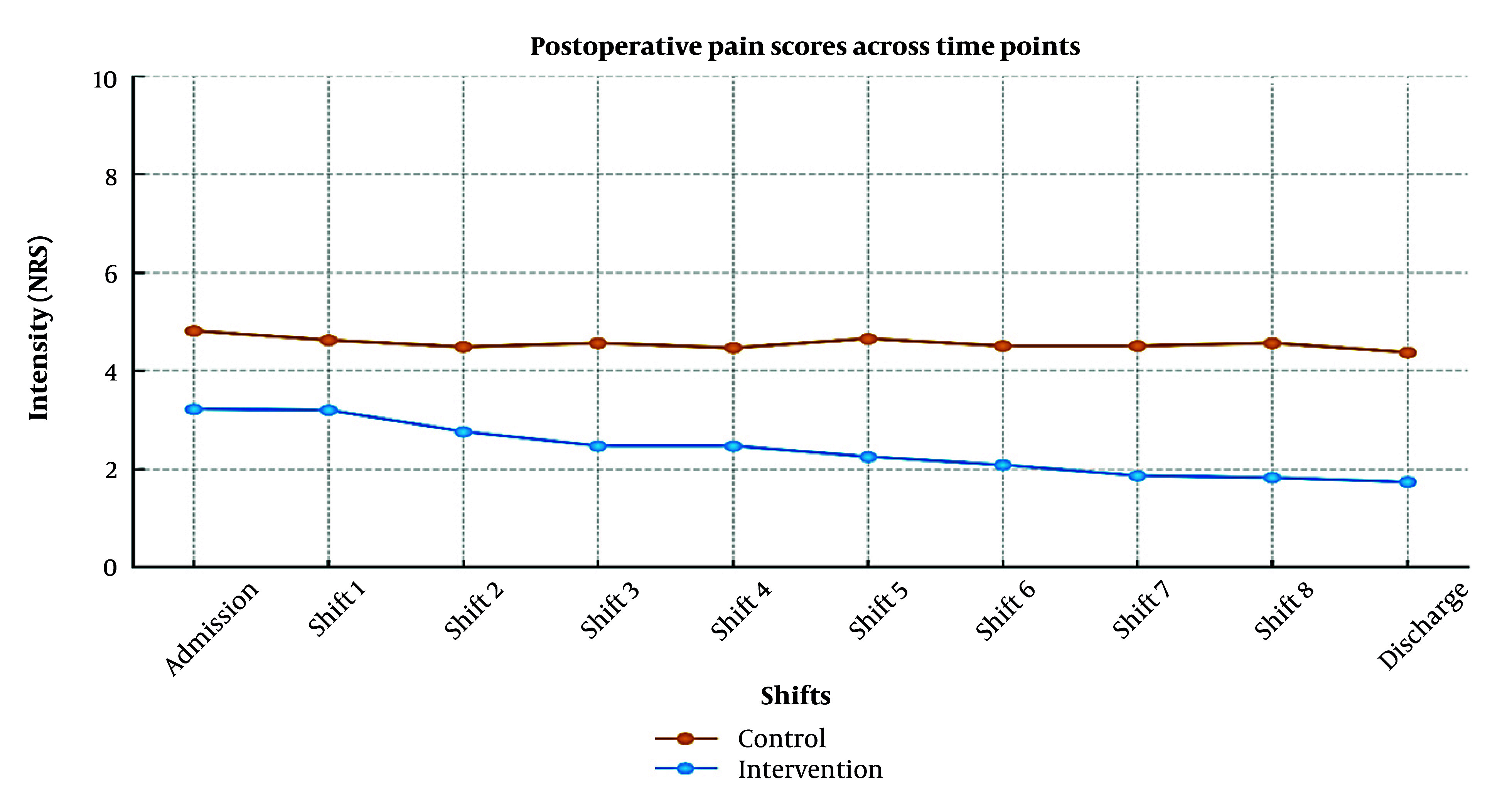
Postoperative pain scores (NRS) in control and intervention groups across time points

Postoperative pain intensity, measured using the 0 - 10 NRS, was significantly lower in the aromatherapy group at all assessment points from ICU admission to hospital discharge (Figure 3). At ICU admission, the intervention group reported reduced pain (mean 3.22 ± 1.22 vs. 4.81 ± 1.09; Z = -5.89, P < 0.001), yielding a large effect size (Cohen’s d = 1.35, 95% CI: 1.02 - 1.68). This difference persisted through discharge (1.73 ± 1.10 vs. 4.37 ± 1.36; Z = −7.50, P < 0.001; d = 2.13, 95% CI: 1.75 - 2.51), representing a 2.64-point mean reduction, substantially exceeding the minimal clinically important difference (MCID) for pain after cardiac surgery (≥ 1.5 points) (26).

**Figure 4.**
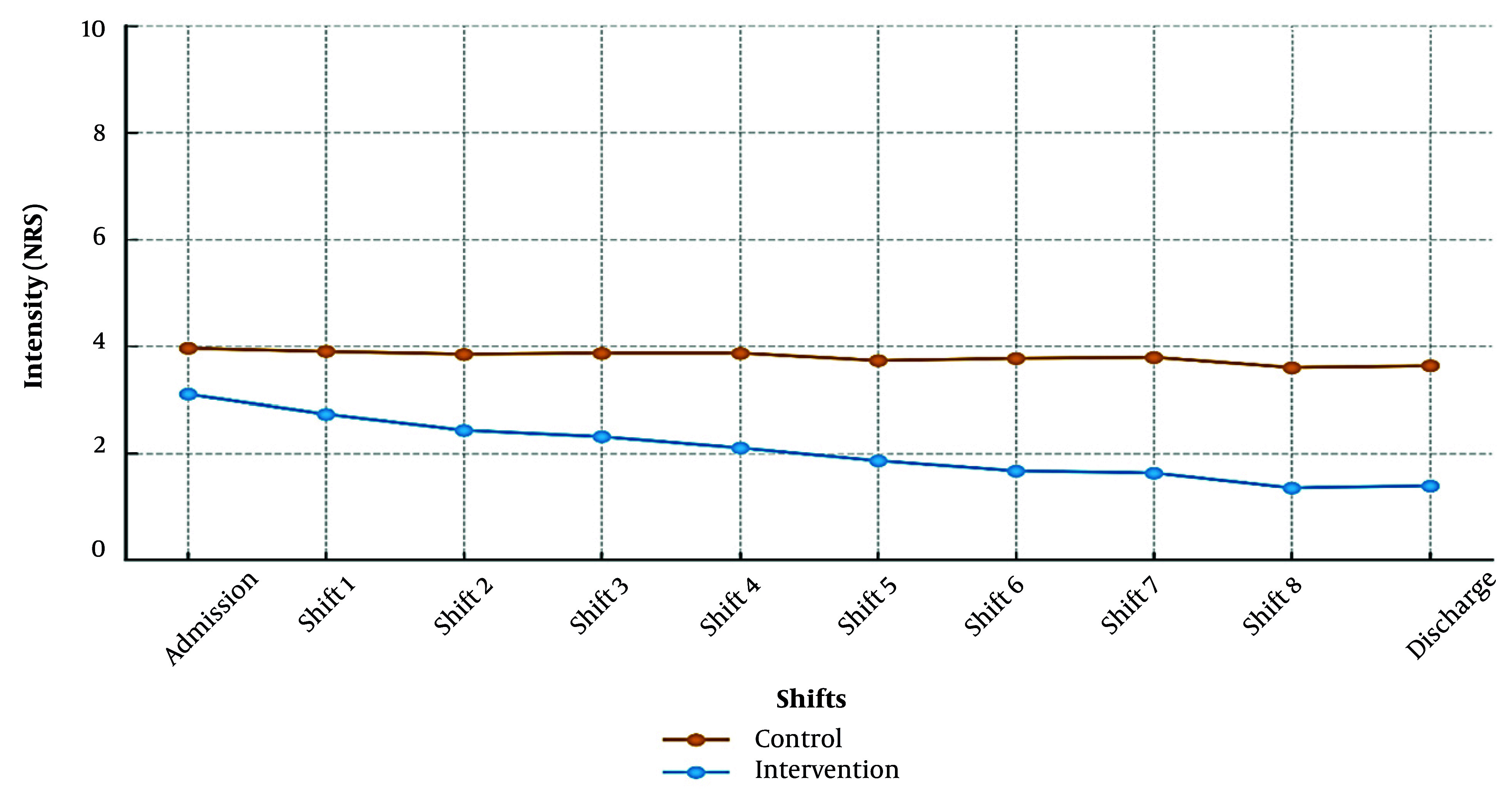
Postoperative nausea and vomiting (PONV) scores (NRS) in control and intervention groups across time points

Similarly, PONV severity was significantly attenuated in the intervention group from admission (3.10 ± 1.46 vs. 3.96 ± 1.34; Z = -2.99, P = 0.003; d = 0.60, 95% CI: 0.32 - 0.88) to discharge (1.39 ± 1.65 vs. 3.63 ± 1.33; Z = -6.07, P < 0.001; d = 1.52, 95% CI: 1.18 - 1.86). The 2.24-point NRS reduction at discharge exceeds the PONV MCID threshold of 1.3 points (Figure 4). Consistent with these findings, the intervention group required fewer rescue antiemetics (P = 0.03 - 0.04) and experienced shorter nausea duration (P < 0.001). All effect sizes were calculated using pooled standard deviations, validated for non-normal data with balanced group sizes (n₁ = 52, n₂ = 51).

The incidence of postoperative delirium, operationalized as an ICDSC total score ≥ 4 and confirmed by CAM-ICU, was markedly lower in the intervention group at 24 hours (28.8% vs. 11.8%; χ² = 4.12, P = 0.042), 48 hours (34.6% vs. 15.7%; χ² = 4.85, P = 0.028), and 72 hours (23.1% vs. 7.8%; χ² = 4.02, P = 0.045) post-surgery (Table 2), yielding absolute risk reductions of 17.0%, 18.9%, and 15.3%, respectively.

**Table 2.** Incidence of Postoperative Delirium (ICDSC ≥ 4) in Control and Intervention Groups at 24-, 48-, and 72-Hours Post-Surgery ^a^

Time Point (h)	Control (n = 52)	Intervention (n = 51)	χ²	P-Value
**24**	15 (28.8)	6 (11.8)	4.12	0.042
**48**	18 (34.6)	8 (15.7)	4.85	0.028
**72**	12 (23.1)	4 (7.8)	4.02	0.045

^a^ Values are expressed as No. (%).

Subscale analyses of the ICDSC (Table 3) revealed significant reductions in the intervention group for most domains at 24 and 48 hours, including altered level of consciousness (24h: χ² = 4.31, P = 0.038; 48h: χ² = 7.77, P = 0.005), inattention (24h: χ² = 10.83, P = 0.001; 48h: χ² = 4.16, P = 0.041), disorientation (24h: χ² = 10.40, P = 0.001), inappropriate speech/mood (24h: χ² = 10.46, P = 0.001; 48h: χ² = 5.50, P = 0.019), sleep-wake disturbances (24h: χ² = 8.79, P = 0.003; 48h: χ² = 11.89, P = 0.001), and symptom fluctuations (trending lower, P = 0.465 - 0.097). At 72 hours, differences persisted primarily in inappropriate speech/mood (χ² = 3.86, P = 0.049) and sleep-wake disturbances (χ² = 4.05, P = 0.044), with odds ratios favoring the intervention (e.g., OR = 0.243 for sleep disturbances at 48h, 95% CI 0.107 - 0.552). Hallucinations showed no significant intergroup differences (P = 0.061 - 0.631).

**Table 3.** Comparison of Intensive Care Delirium Screening Checklist Subscale Items Between Control and Intervention Groups Across Time Points ^a^

Time (h)/Subscales/Groups	Yes	No	χ² (P-Value)	OR	95% CI Lower	95% CI Upper
**24**						
Change of consciousness						
Intervention	19 (37.3)	32 (62.7)	4.312 (0.038)	0.435	0.198	0.960
Control	30 (57.7)	22 (42.3)	-	-	-	-
Inattention/Orders						
Intervention	14 (27.5)	37 (72.5)	10.827 (0.001)	0.256	0.112	0.586
Control	31 (59.6)	21 (40.4)	-	-	-	-
Disorientation						
Intervention	7 (13.7)	44 (86.3)	10.399 (0.001)	0.217	0.082	0.572
Control	22 (42.3)	30 (57.7)	-	-	-	-
Hallucinations						
Intervention	3 (5.9)	48 (94.1)	0.231 (0.631)	1.563	0.250	9.765
Control	2 (3.8)	50 (96.2)	-	-	-	-
Agitation						
Intervention	16 (31.4)	35 (68.6)	3.699 (0.054)	0.457	0.205	1.021
Control	26 (50.0)	26 (50.0)	-	-	-	-
Inappropriate speech/mood						
Intervention	10 (19.6)	41 (80.4)	10.461 (0.001)	0.244	0.101	0.588
Control	26 (50.0)	26 (50.0)	-	-	-	-
Sleep-wake disturbance						
Intervention	26 (51.0)	25 (49.0)	8.794 (0.003)	0.279	0.118	0.661
Control	41 (78.8)	11 (21.2)	-	-	-	-
Symptom fluctuation						
Intervention	18 (35.3)	33 (64.7)	0.533 (0.465)	0.744	0.336	1.648
Control	22 (42.3)	30 (57.7)	-	-	-	-
**48**						
Change of consciousness						
Intervention	8 (15.7)	43 (84.3)	7.765 (0.005)	0.275	0.108	0.700
Control	21 (40.4)	31 (59.6)	-	-	-	-
Inattention/Orders						
Intervention	3 (5.9)	48 (94.1)	4.160 (0.041)	0.263	0.068	1.018
Control	10 (19.2)	42 (80.8)	-	-	-	-
Disorientation						
Intervention	3 (5.9)	48 (94.1)	2.437 (0.118)	0.344	0.086	1.378
Control	8 (15.4)	44 (84.6)	-	-	-	-
Hallucinations						
Intervention	1 (2.0)	50 (98.0)	0.324 (0.569)	0.500	0.044	5.692
Control	2 (3.8)	50 (96.2)	-	-	-	-
Agitation						
Intervention	7 (13.7)	44 (86.3)	3.505 (0.061)	0.392	0.145	1.065
Control	15 (28.8)	37 (71.2)	-	-	-	-
Inappropriate speech/mood						
Intervention	4 (7.8)	47 (92.2)	5.500 (0.019)	0.255	0.077	0.846
Control	13 (25.0)	39 (75.0)	-	-	-	-
Sleep-wake disturbance						
Intervention	17 (33.3)	34 (66.7)	11.889 (0.001)	0.243	0.107	0.552
Control	35 (67.3)	17 (32.7)	-	-	-	-
Symptom fluctuation						
Intervention	4 (7.8)	47 (92.2)	0.401 (0.527)	0.652	0.173	2.464
Control	6 (11.5)	46 (88.5)	-	-	-	-
**72**						
Change of consciousness						
Intervention	0 (0)	51 (100)	0.990 (0.320)	-	-	-
Control	1 (1.9)	51 (98.1)	-	-	-	-
Inattention/Orders						
Intervention	0 (0)	51 (100)	0.990 (0.320)	-	-	-
Control	1 (1.9)	51 (98.1)	-	-	-	-
Disorientation						
Intervention	0 (0)	51 (100)	2.000 (0.157)	-	-	-
Control	2 (3.8)	50 (96.2)	-	-	-	-
Hallucinations						
Intervention	0 (0)	51 (100)				
Control	0 (0)	52 (100)	-	-	-	-
Agitation						
Intervention	0 (0)	51 (100)	0.990 (0.320)	-	-	-
Control	1 (1.9)	51 (98.1)	-	-	-	-
Inappropriate speech/mood						
Intervention	2 (3.9)	49 (96.1)	3.859 (0.049)	0.224	0.045	1.114
Control	8 (15.4)	44 (84.6)	-	-	-	-
Sleep-wake disturbance						
Intervention	8 (15.7)	43 (84.3)	4.051 (0.044)	0.383	0.148	0.992
Control	17 (32.7)	35 (67.3)	-	-	-	-
Symptom fluctuation						
Intervention	1 (2.0)	50 (98.0)	2.750 (0.097)	0.188	0.021	1.669
Control	5 (9.6)	47 (90.4)	-	-	-	-

^a^ Values are expressed as No. (%).

### 4.2. Secondary Outcomes

Baseline inflammatory biomarker levels were comparable between groups (Table 4). At 72 hours, the NLR was significantly lower in the intervention group (3.05 ± 1.50) compared with the control group (3.94 ± 0.99) (Z = 3.93, P < 0.001). Similarly, CRP concentrations at 72 hours were significantly reduced in the aromatherapy group (P < 0.001), indicating attenuation of the postoperative inflammatory response. No adverse events attributable to the intervention were reported, confirming a favorable safety profile.

**Table 4.** Comparison of Neutrophil-to-Lymphocyte Ratio and C-Reactive Protein in Control and Intervention Groups ^a^

Biomarker and Time Point	Control (n = 52)	Intervention (n = 51)	Mann-Whitney U (Z)	P-Value
**NLR**				
Baseline (admission)	4.50 ± 1.01/4.50 (1.60)	4.68 ± 2.43/3.90 (2.70)	-1.00	0.316
72 h post-admission	3.94 ± 0.99/4.05 (1.60)	3.05 ± 1.50/2.60 (2.00)	3.93	< 0.001
**CRP (mg/L)**				
Baseline (admission)	11.29 ± 3.78/10.95 (4.00)	10.16 ± 6.47/10.00 (4.00)	-2.45	0.014
72 h post-admission	9.82 ± 3.47/9.90 (4.00)	6.80 ± 3.82/6.20 (5.00)	-4.29	< 0.001

^a^ Values are expressed as mean ± SD/median (IQR).

## 5. Discussion

This randomized controlled trial demonstrates that inhalation aromatherapy with lemon essential oil significantly improves several important postoperative outcomes in patients undergoing CABG. In particular, patients who received lemon aromatherapy experienced reductions in postoperative pain, PONV, delirium, and inflammatory biomarkers. Despite randomization, baseline imbalances existed in prior hospitalization and prior ICU admission. Per our prespecified Statistical Analysis Plan, multivariable logistic regression adjusting for these covariates plus age and CPB duration confirmed robust intervention effects, with no meaningful attenuation of effect estimates. Similarly, analysis of covariance (ANCOVA) models demonstrated persistent reductions in CRP/NLR after covariate adjustment. Groups remained well-balanced in current severity markers (ASA class, surgery duration, comorbidities), supporting causal interpretation.

Specifically, we observed meaningful reductions in pain intensity (up to -2.64 at discharge), PONV severity and frequency (up to -2.24), and delirium incidence (up to 21% absolute risk reduction at 48 hours), alongside attenuated inflammatory responses as evidenced by lower NLR and CRP levels at 72 hours post-admission. These findings are consistent with a growing body of evidence supporting complementary interventions in perioperative care, particularly in high-risk cardiac populations where inflammation and neurocognitive disturbance frequently hinder recovery (10, 13).

Emerging evidence suggests that the analgesic properties of lemon essential oil may involve modulation of central inhibitory pathways, including GABAergic and opioid receptor activity (15). In animal models, eriocitrin — a major lemon-derived flavonoid — has demonstrated significant antinociceptive effects in postoperative pain models (20). Consistent with these findings, systematic reviews and meta-analyses have shown that several forms of aromatherapy can effectively reduce postoperative pain (27). Accumulating evidence also suggests that aromatherapy — whether administered by inhalation or topical application — may alleviate various types of pain, including post-spinal headache following dural puncture (28).

Our results on pain and PONV mitigation closely mirror those from a parallel randomized controlled trial by Rambod et al., who reported comparable NRS decrements in pain and nausea/vomiting severity among 90 patients post-lower extremity fracture surgery, with reduced antiemetic needs (15). The temporal dosing similarity likely contributed to these concordant effects, underscoring lemon aromatherapy's feasibility across surgical contexts. Unlike previous studies that did not evaluate or demonstrate effects on neurocognitive outcomes, our trial extends these benefits to delirium prevention, which is particularly relevant in CABG patients (29). This difference may be attributable to the cardiac-specific protocol used in our study, the incorporation of ICU-based administration methods, and the use of validated delirium screening tools, highlighting essential oil's broader neuroprotective potential via sleep-wake stabilization and psychomotor modulation (30).

Mechanistically, the observed reductions in CRP and NLR are consistent with preclinical findings showing that lemon-derived compounds modulate inflammatory pathways by inhibiting NF-κB and related cytokine cascades (e.g., IL-6, TNF-α) (19, 22). In the context of CABG, where CPB-induced inflammation contributes to delirium and PONV (8, 11), these biomarkers may represent plausible intermediaries linking aromatherapy to improved clinical outcomes; our 72-hour decrements suggest lemon modulates the hypothalamic-pituitary-adrenal axis, curbing oxidative stress and hypothalamic-pituitary-adrenal hyperactivity that perpetuate postoperative cognitive dysfunction (20, 21).

Beyond its analgesic potential, inhaled lemon essential oil has been shown to alleviate anxiety in both healthy individuals and cardiac patients, possibly due to its anti-inflammatory actions, inhibition of prostaglandin synthesis, and modulation of oxidative stress pathways (31). This is further supported by subscale analyses showing preferential improvements in sleep disturbances and symptom fluctuations, similar to peppermint aromatherapy's delirium-lowering effects in orthopedic elders (14), though our trial uniquely ties these to quantifiable inflammatory shifts absent in prior aromatherapy studies.

Notably, our delirium findings diverge from some cardiac cohorts, where aromatherapy yields inconsistent neurocognitive benefits (13, 32). For instance, while Rambod et al. noted anxiolytic effects in myocardial infarction without delirium assessment (13), our CABG sample and blinded ICU evaluations may explain the stronger signal. Conversely, alignment with yoga's biomarker reductions in cardiac surgery meta-analyses (for IL-6/CRP) reinforces non-pharmacological strategies' role in frailty-prone patients (3, 10). These consistencies affirm lemon's safety profile, with no adverse events reported, positioning it as a cost-effective alternative to opioids, which cause nausea and vomiting in over 30% of cases (9, 12).

Limitations temper these insights. As a single-center trial at Baqiyatallah University, generalizability may be constrained by regional demographics and standardized protocols; multicenter replication is warranted. While the single-center design limits generalizability, it was deliberately chosen to minimize implementation bias when addressing the multifactorial pathophysiology of postoperative delirium. Although bitter almond oil is not completely odorless, its minimal scent and the triple-blind design with identical administration limited the risk of unblinding. Future studies should explore dose-response (e.g., varying limonene concentrations), long-term cognitive outcomes (> 72 hours), and integration with multimodal protocols like enhanced recovery after surgery (11).

In conclusion, lemon inhalation aromatherapy emerges as a simple, efficacious intervention that not only alleviates pain and PONV but also safeguards against delirium in CABG patients, potentially via blunted inflammatory cascades. By bridging gaps in prior fracture-focused trials (15, 16), these results advocate for its routine perioperative adoption, promising enhanced recovery and reduced healthcare burdens in an era of aging CAD epidemics (1, 5). Rigorous, diverse trials will solidify its place in evidence-based cardiac care. Collectively, these findings highlight the multifaceted mechanisms through which lemon-derived compounds may contribute to pain relief and psychological well-being.

### 5.1. Conclusions

This study provides robust evidence that inhalation aromatherapy with lemon essential oil (*C. limon*) exerts multifaceted therapeutic effects in the postoperative management of CABG patients. From a translational standpoint, the intervention's non-invasive profile, devoid of adverse events and compatible with standard ICU protocols, positions it as a pragmatic adjunct to pharmacological regimens, potentially curtailing opioid reliance, expediting recovery trajectories, and alleviating the socioeconomic encumbrance of prolonged hospitalizations (estimated 2.2-day extension per delirium case). Limitations include single-center design, baseline imbalances in cardiovascular risk factors, and lack of long-term cognitive follow-up. Future multicenter trials should explore dose-response relationships and long-term cognitive outcomes. Lemon inhalation aromatherapy presents a safe, low-cost adjunct to standard postoperative care that significantly improves multiple recovery domains following CABG surgery. Given the high prevalence and clinical impact of postoperative delirium, pain, and PONV, and the absence of adverse effects, these findings support the incorporation of aromatherapy into evidence-based perioperative pathways for cardiac surgery patients.

## Data Availability

The raw data and materials are available from the corresponding author upon reasonable request.

## Clinical Trial Registration Code:

IRCT20231021059799N2.
